# An Extraordinary Case of Hemorrhage

**Published:** 1853-10

**Authors:** 


*An Extraordinary Case of Hemorrhage*-—T. C. Harris Esq., of London, gives a case of hemorrhage, after the removal of a superior molar tooth. His mode of treatment was, to take an impression of that part of the mouth, and adjust a silver plate with clasps, to the adjoining teeth. The alveolus is plugged up with cotton, dipped in benzoin; the plate is then applied, and the clasps closed on the teeth; a cork is applied over the silver plate, so that when the mouth is closed, pressure will be applied, and thus stop the hemorrhage. We have often accomplished the same thing, by merely bending a piece of silver or gold plate, forming a kind of saddle, to embrace the gum around the socket; then plug up the socket, applying our plate, with a piece of soft leather underneath, to prevent the plate from hurting the gum; then adapt over this, a piece of cork or pledgets of cotton; close the mouth to obtain pressure, and keep it closed by a bandadge. If the alveolus is somewhat fractured, the plug of cotton would force it open, and would not arrest the bleeding. The ends of the silver plate, force together the gum and walls of the broken socket, and thus mantain the parts, in the best possible position, to arrest the hemorrhage.

We do not regard cases of hemorrhage, which can be arrested by compress, as giving any very particular difficulty. Almost any man of mechanical tact, would soon plug up the socket, and apply an efficient compress; and these cases we do not doubt, often occur, without any very special hemorrhagic diathesis. The artery going to the tooth, may be larger than usual, there may be a congested state of the blood vessels, from long continued inflammation, &c. These things may exist, and hemorrhage take place, after the most careful extraction of the teeth. When a marked hemorrhagic diathesis exists, we do not believe that compression is effectual. In one family of this city, there are four members, all of whom exhibit this diathesis, and in whom compression has always failed to arrest the hemorrhage. The actual cautery will not arrest for more than a few hours. In these cases, we have always had to resort to constitutional treatment. We have had three of these individuals under our care, one of them twice, and the hemorrhage has generally lasted from two to four days.

In the July No, of the Dental News Letter, a case is given, resulting ultimately in death. In this case the hemorrhage had been arrested, and after he had for a few days, been rapidly recovering, hemorrhage again ensued, and proved fatal. In the April No. of the American Journal of Dental Science, we have a reported case, treated by Dr. F. L. Crane, of Easton, Pa., where the same contrivance of plate and appliance, as is recommended by F. C. Harris Esq., was effectual in arresting, a very difficult case of hemorrhage, after every other means had failed.

Dr. Saltenstall, of Columbus, Miss., gives a somewhat similar case. (See Oct. No. of Journal for '52.)

